# Effect of Red Blood Cell Storage Duration on Cerebral Blood Oxygenation in Elective Cranial Surgery: A Prospective Randomized Controlled Study

**DOI:** 10.7759/cureus.86351

**Published:** 2025-06-19

**Authors:** Anjali Kumari, Brijesh P Singh, Ravi Prakash, Hemlata Hemlata, Dinesh Singh, Monica Kohli

**Affiliations:** 1 Anaesthesiology, King George's Medical University, Lucknow, IND

**Keywords:** blood transfusion, cerebral oxygenation, jugular venous saturation, neurological outcome, neurosurgery, prbc storage lesion, randomised controlled trial

## Abstract

Background: The clinical significance of storage-related changes in packed red blood cells (PRBCs), termed “storage lesions,” remains controversial. These biochemical and structural alterations may affect oxygen delivery, particularly in patients undergoing neurosurgical procedures, where cerebral oxygenation is critical. This study aimed to evaluate the effect of PRBC storage duration on cerebral oxygenation and neurological outcomes in patients undergoing elective neuro-oncological surgery.

Methods: In this prospective, double-blind, randomized controlled trial, 80 adult patients (American Society of Anesthesiologists (ASA) physical status I-II) scheduled for elective neuro-oncological surgery were enrolled and randomized into two groups: Group N (PRBCs stored <14 days) and Group O (PRBCs stored >14 days). Standard general anesthesia protocols were followed. Jugular bulb catheters were placed for serial monitoring of cerebral oxygenation parameters (saturation of jugular venous oxygen (SjvO₂), partial pressure of jugular venous oxygen (PjvO₂), lactate, and potential of hydrogen (pH)). Hemodynamic and blood gas values were recorded at baseline and hourly intraoperatively. Neurological outcomes were assessed using the Glasgow Outcome Scale-Extended (GOSE) scores at hospital discharge.

Results: Demographic characteristics, intraoperative parameters (surgery duration, blood loss), and transfusion volumes were comparable between groups. Hemodynamic variables and arterial/jugular venous blood gas measurements showed no consistent or clinically significant differences. A transient increase in SjvO₂ was observed in Group O at one hour post transfusion (p < 0.05), but this was not sustained. There were no statistically significant differences in GOSE scores or ICU and hospital stay durations between groups.

Conclusion: The duration of PRBC storage did not significantly affect intraoperative cerebral oxygenation or early postoperative neurological outcomes in patients undergoing neuro-oncological surgery. These findings suggest that transfusion of older PRBCs (>14 days) is clinically safe in this setting.

## Introduction

Blood loss in neuro-oncological surgeries can range from minimal to massive, depending on several factors such as the tumor's type, grade, size, vascularity, proximity to major cranial vessels and venous sinuses, and the length of the surgery [1,2], Studies have established that anemia is an independent risk factor for increased postoperative morbidity and mortality in neurosurgical patients [3]. In the early stages of anemia, the brain compensates by dilating blood vessels to ensure adequate oxygen delivery. However, in the case of brain injury, these compensatory mechanisms are impaired, leading to further oxygen deficiency at the cellular level due to reduced hemoglobin [4]. Evidence supports that blood transfusion can enhance cerebral metabolism and oxygenation [5]. Transfusions help counteract the reduction in oxygen delivery (DO2), while the cerebral circulation increases the oxygen extraction fraction (OEF), ensuring sufficient oxygen for cellular metabolism (DO2×OEF) and preventing ischemia, which would otherwise reduce the cerebral metabolic rate of oxygen (CMRO2) [6], The duration of packed red blood cell (PRBC) storage may affect their oxygen affinity, shape flexibility, membrane stability, function, and overall transfusion effectiveness.

The term "storage lesion" refers to the complex biochemical and biomechanical changes that occur during PRBC storage outside the body, altering both the PRBCs' properties and the surrounding plasma and causing numerous systemic adverse effects over the body [7-11]. The clinical significance of storage lesions in PRBCs remains debated, with no clear consensus on the threshold that differentiates fresh from aged PRBCs. Moreover, there are conflicting findings regarding the relationship between PRBC storage duration and the rates of morbidity and mortality following transfusion. The majority of available studies are of a retrospective design. The existing literature also contains several flaws, including a lack of strict criteria, variability in study methods and patient groups, inconsistent outcome measures, and the use of varying thresholds to define the age of PRBCs, ranging from five to 27 days [12-14].

Most of the previous studies have shown the impact of the storage duration of RBCs in critically ill patients in ICU settings and traumatic brain injury patients [15,16]. There is a paucity of literature about the effect of storage duration of PRBCs on the outcome of patients undergoing neuro-oncological surgeries. We propose a hypothesis that newer blood is better than older blood for cerebral oxygenation. This prospective randomized study was an effort to find out the effect of the duration of storage of PRBC on cerebral oxygenation and neurological outcome in patients who underwent elective neuro-oncological surgeries.

## Materials and methods

This prospective, double-blind, randomized controlled trial was conducted at the Department of Anesthesiology & Critical Care, King George’s Medical University (KGMU), Lucknow, India, between January 2023 and March 2024. The study received approval from the Institutional Ethics Committee (IEC/160/Ethics/2023) and was registered with the Clinical Trials Registry of India (CTRI/2023/08/056928). Written informed consent was obtained from all participants prior to enrollment.

The sample size was determined based on data from a previous study by Yamel et al., which reported a mean difference of 5% in post-transfusion jugular venous oxygen saturation (SjvO₂) between groups, with a standard deviation of 8% [7]. 

The formula used is as follows:

$N = \frac{2(Z_1 + Z_2)^2 \cdot SD^2}{d^2}$

where Z₁ = 1.96 (for 95% confidence), Z₂ = 0.842 (for 80% power), SD = 8, and d = 5, the required sample size was 80 patients (40 per group).

Eighty patients aged 18-60 years, of either sex, with an American Society of Anesthesiologists (ASA) physical status I-II, anticipated intraoperative blood transfusion, and scheduled for elective neuro-oncological surgery under general anesthesia were enrolled. Patients not requiring intraoperative blood transfusion were excluded from the study. Exclusion criteria included a history of psychiatric or neurological disorders, pregnancy or lactation, previous craniotomy or preoperative blood transfusion, and refusal to provide informed consent. Patients were randomly assigned to two groups using a computer-generated random number sequence. Group allocation was concealed in sequentially numbered, opaque, sealed envelopes, which were opened on the day of surgery. Participants, attending anesthesiologists, and neurosurgeons were blinded to group assignments. The blood bank was informed to provide blood and blood products with group-specific storage durations previously. The two groups were as followes: Group N (new blood): received PRBCs stored for less than or equal to 14 days; Group O (old blood): received PRBCs stored for more than 14 days.

Standard ASA monitoring (ECG, non-invasive blood pressure (NIBP), peripheral oxygen saturation (SpO₂), end-tidal carbon dioxide (EtCO₂), and temperature) was initiated. Intravenous access was established, and general anesthesia was induced with fentanyl (2 μg/kg), propofol (2 mg/kg), and vecuronium (0.1 mg/kg). After three minutes, laryngoscopy and endotracheal intubation were performed using an appropriately sized tube. Anesthesia was maintained with a mixture of 40% oxygen and 60% air, using sevoflurane (1.5%-2.0% minimum alveolar concentration (MAC)), along with continuous infusions of fentanyl (1 μg/kg/hr) and vecuronium (0.03 mg/kg/hr). Following induction, two central venous catheters (CVCs) were inserted into the right internal jugular vein under ultrasound guidance. One catheter was placed in the conventional antegrade direction for central venous pressure (CVP) monitoring, fluid resuscitation, and vasopressor administration. The second catheter was introduced in a retrograde fashion, with the tip positioned in the jugular bulb, confirmed intraoperatively using C-arm fluoroscopy. This catheter was used for sampling jugular venous blood to assess saturation of jugular venous oxygen (SjvO₂) and other cerebral oxygenation parameters. Blood samples were withdrawn slowly (≤2 mL/min) to avoid contamination from extracranial sources. The blood bank was informed beforehand to ensure the availability of PRBC as per the allotted group.

Intraoperatively, when a transfusion was indicated based on clinical judgment (e.g., blood loss, hemoglobin threshold), patients received leukoreduced PRBC units according to their group assignment (Group “N” or Group “O”). The date of collection and expiry of the unit was concealed by applying an opaque sticker on it. The total number of PRBC units and additional blood products (fresh frozen plasma (FFP) and platelets) administered was recorded. Any transfusion reactions were promptly treated and documented. Neuromuscular blockade was reversed using neostigmine (0.05 mg/kg) and glycopyrrolate (0.01 mg/kg). Extubation was performed once the patient demonstrated adequate neuromuscular recovery and obeyed verbal commands. The decision for elective postoperative ventilation was made collaboratively by the attending anesthesiologist and neurosurgeon based on clinical parameters.

The primary outcome was change in cerebral oxygenation parameters, especially SjvO₂, after PRBC transfusion, while secondary outcomes included hemodynamic parameters (systolic blood pressure (SBP), diastolic blood pressure (DBP), mean arterial pressure (MAP), and central venous pressure (CVP), temperature, urine output, peripheral and cerebral oxygenation (SpO₂, oxygen saturation (SaO₂), partial pressure of oxygen in arterial blood (PaO₂), partial pressure of carbon dioxide in arterial blood (PaCO₂), partial pressure of oxygen in mixed venous blood (PvO₂), mixed venous oxygen saturation (SvO₂), partial pressure of jugular venous oxygen (PjvO₂), SjvO₂, potential of hydrogen (pH), bicarbonate (HCO₃)), lactate, base deficit, neurological outcome (assessed using Glasgow Outcome Scale-Extended (GOSE) at hospital discharge), and duration of ICU and hospital stay. All physiological parameters and laboratory values were recorded at baseline (T0) and hourly after the first PRBC transfusion until the end of surgery as T1, T2, T3, and so on.

Statistical analysis

Data were entered into Microsoft Excel and analyzed using IBM SPSS version 25.0 (IBM Corp., Armonk, NY, USA). Continuous variables were expressed as mean ± standard deviation and compared using Student’s t-test. Categorical variables were compared using the chi-square or Fisher’s exact test. A p-value < 0.05 was considered statistically significant.

## Results

Study population

Eighty patients undergoing elective neurosurgery were enrolled in this prospective, randomized controlled study and equally allocated to two groups (n=40 each). Group N received transfusions with red blood cells stored for less than or equal to 14 days (“new blood”), while Group O received blood stored for more than 14 days (“old blood”). All patients enrolled in the study received blood transfusions.

Baseline characteristics

Baseline demographic and clinical characteristics were comparable between the two groups (Table 1). The mean age of participants in Group N was 36.75 years (± SD), slightly lower than the mean age in Group O (40.45 years), though the difference was not statistically significant (t = -1.367, p = 0.176). Gender distribution was also balanced, with Group N comprising 40% females and 60% males, while Group O comprised 52.5% females and 47.5% males (p = 0.262). There were no significant differences in mean body weight (Group N: 58.85 kg vs. Group O: 61.55 kg, p = 0.143) or height (Group N: 160.08 cm vs. Group O: 158.55 cm, p = 0.315). ASA physical status classification showed an even distribution in Group N (ASA I: 50%, ASA II: 50%), while Group O had a higher proportion of ASA II patients (62.5%) compared to ASA I (37.5%). However, this difference was not statistically significant (p = 0.260) (Table 1).

**Table 1:** Baseline demographics and clinical characteristics of the study group ASA: American Society of Anesthesiologists; PRBC: packed red blood cells; FFP: fresh frozen plasma; GOSE: Glasgow Outcome Scale-Extended

Demographic	Group N (n=40)	Group O (n=40)	Test	Remarks
Gender				
Male	24	21	Chi-square test	χ=1.257 P=0.262
Female	16	19		
Age (years)	36.75±11.96	40.45±12.25	Student's t-test	t=-1.367 p=0.176
Height (cm)	160.08±6.72	158.55±6.77	Student's t-test	t=1.011 p=0.315
Weight (kg)	58.85±8.18	61.55±8.15	Student's t-test	t=-1.479 p=0.143
ASA Grade				
I	20	15	Chi-square test	χ=1.270 P=0.260
II	20	25		
Duration of surgery (hours)	6.99±1.17	6.88±1.24	Student's t-test	t= 0.092 p= 0.927
Blood loss (ml)	660.25±262.07	681.00±274.27	Student's t-test	t= -0.346 p= 0.730
Transfusion				
PRBC (units)	1.70±0.79	1.98±0.08	Student's t-test	t= -1.546 p= 0.126
FFP (units)	0.65±0.89	0.98±0.89	Student's t-test	t= -1.629 p= 0.107
Platelets (units)	0.30±0.61	0.27±0.60	Student's t-test	t= 0.185 p= 0.853
ICU stay (days)	1.3±4.6	2.2±5.6	Student's t-test	t= -0.761 p= 0.449
Hospital stay (days)	29.7±7.6	32.5±10.1	Student-t test	t= -2.813 p= 0.165
GOSE score	7.7±0.56	7.65±0.70	Student-t test	t= 0.235 p= 0.815

Intraoperative characteristics

The duration of surgery and estimated blood loss were statistically similar between the groups. Mean operative time was 6.90 ± 1.17 hours in Group N and 6.88 ± 1.24 hours in Group O (p = 0.927). Average intraoperative blood loss was also comparable: 660.25 ± 262.07 ml in Group N versus 681.00 ± 274.27 ml in Group O (p = 0.730). The quantity of blood products transfused intraoperatively showed no statistically significant differences. The mean number of PRBC units transfused was 1.70 ± 0.79 in Group N and 1.98 ± 0.08 in Group O (p = 0.126). Similarly, the use of FFP and platelets did not differ significantly between the groups (FFP: p = 0.107; platelets: p = 0.853).

Hemodynamic parameters

Hemodynamic variables, including heart rate (HR), SBP and DBP, MAP, and CVP, were recorded at multiple intervals (T1-T9). No statistically significant intergroup differences were found across all time points (p > 0.05 for all comparisons). Group N and Group O showed nearly identical trends in cardiovascular stability throughout the perioperative period.

A significant intergroup difference in mean body temperature was observed at time interval T8. Group N had a mean temperature of 35.7°C (± 0.8), while Group O had a significantly higher value of 36.3°C (± 0.4; p = 0.041). No other time intervals showed significant temperature variation between the groups.

Arterial blood gas parameters

Analysis of arterial blood gases at time points T1 through T4 revealed no significant differences between the groups in pH, PaO₂, PaCO₂, HCO₃⁻, lactate, or base deficit. While the arterial pH showed a near-significant trend at T3 (p = 0.102), overall, gas exchange and acid-base status remained comparable throughout the study (p > 0.05 across all parameters) (Table 2).

**Table 2:** Intergroup comparison of arterial blood gas values pH: potential of hydrogen; SaO_2_: oxygen saturation; PaO_2_: partial pressure of oxygen in arterial blood; PaCO_2_: partial pressure of carbon dioxide in arterial blood; HCO3: bicarbonate; BD: base deficit; T: time after surgery (hourly); Group N: new blood, received PRBCs stored for less than or equal to 14 days; Group O: old blood, received PRBCs stored for more than 14 days; PRBCs: packed red blood cells

Variable			T1	T2	T3	T4
pH	Group N	Mean±SD	7.35±0.05	7.31±0.88	7.32±0.05	7.34±0.01
	Group O	Mean±SD	7.37±0.07	7.36±0.05	7.34±0.04	7.36±0.03
	Student’s t-test	t	-0.865	-1.541	-1.697	-1.204
		P	0.39	0.127	0.102	0.263
SaO_2_	Group N	Mean±SD	98.8±0.9	98.8±0.9	98.9±0.8	99.3±0.2
	Group O	Mean±SD	98.9±0.9	98.7±1.1	99.0±0.7	99.3±0.2
	Student’s t-test	t	-0.544	0.656	-0.359	0.306
		P	0.588	0.514	0.723	0.768
PaO_2_	Group N	Mean±SD	200.2±107.4	162.4±65.1	160.1±36.5	172.0±58.4
	Group O	Mean±SD	193.0±83.9	161.1±60.2	164.3±51.7	186.2±76.1
	Student’s t-test	t	0.332	0.091	-0.248	-0.306
		P	0.741	0.928	0.806	0.768
PaCO_2_	Group N	Mean±SD	35.0±4.2	32.8±3.2	32.3±2.5	33.5±0.5
	Group O	Mean±SD	35.7±4.3	34.1±3.5	33.5±2.1	33.1±0.9
	Student’s t-test	t	-0.695	-1.715	-1.329	0.679
		P	0.489	0.09	0.196	0.519
HCO3	Group N	Mean±SD	21.2±2.9	20.6±3.0	20.0±3.0	22.2±3.6
	Group O	Mean±SD	21.1±3.2	20.3±3.1	19.5±3.2	21.4±4.1
	Student’s t-test	t	0.07	0.351	0.419	0.304
		P	0.944	0.726	0.679	0.77
Lactate	Group N	Mean±SD	1.7±0.7	2.1±0.9	2.0±0.4	2.4±0.5
	Group O	Mean±SD	1.8±0.9	2.2±1.1	2.2±0.8	1.8±0.6
	Student’s t-test	t	-0.372	-0.435	-0.728	1.526
		P	0.711	0.665	0.473	0.171
BD	Group N	Mean±SD	-2.6±3.4	-4.8±2.3	-5.7±1.2	-6.0±0.4
	Group O	Mean±SD	2.7±3.4	-4.8±2.3	-5.8±1.1	-5.9±0.8
	Student’s t-test	t	0.248	-0.063	0.336	-0.279
		P	0.805	0.95	0.739	0.788

Jugular venous blood gas parameters

Jugular venous sampling revealed one significant difference in jugular venous pH at T2, where Group N showed a higher mean pH than Group O (p = 0.007), suggesting a transient alteration in cerebral acid-base equilibrium. Other parameters, including jugular HCO₃⁻, lactate, base deficit, and PjvO₂ and PjvCO₂, were similar across time points (p > 0.05) (Table 3).

**Table 3:** Intergroup comparison of jugular venous gas values pH: potential of hydrogen; SjvO₂: saturation of jugular venous oxygen; PjvO_2_: partial pressure of jugular venous oxygen; HCO3: bicarbonate; BD: base deficit; T: time after surgery (hourly); Group N: new blood, received PRBCs stored for less than or equal to 14 days; Group O: old blood, received PRBCs stored for more than 14 days; PRBCs: packed red blood cells

Variables			T1	T2	T3	T4
pH	Group N	Mean±SD	7.34±0.04	7.34±0.04	7.32±0.04	7.34±0.01
	Group O	Mean±SD	7.35±0.07	7.31±0.05	7.33±0.04	7.31±0.04
	Student’s t-test	t	-0.571	2.757	-0.167	0.598
		P	0.57	0.007	0.869	0.567
SjVO_2_	Group N	Mean±SD	69.1±7.0	73.2±8.7	77.3±8.2	76.8±6.6
	Group O	Mean±SD	70.7±8	74.8±9.3	73.6±7	75.2±5.3
	Student’s t-test	t	-0.966	-0.818	1.287	0.416
		P	0.337	0.416	0.209	0.688
PjVO_2_	Group N	Mean±SD	40.7±5.8	37.4±4.9	36.9±4.3	37.2±2.9
	Group O	Mean±SD	42.8±8	42.2±17.6	39.2±5.5	36.5±4
	Student’s t-test	t	-1.328	-1.658	-1.225	0.324
		P	0.188	0.101	0.231	0.754
PjvCO_2_	Group N	Mean±SD	42.7±3	41.2±4.6	38.8±4.7	37.5±1.4
	Group O	Mean±SD	42.2±4	40.6±4.3	40±4.5	37.6±2.7
	Student’s t-test	t	0.624	0.577	-0.689	-0.089
		P	0.535	0.565	0.497	0.931
HCO3	Group N	Mean±SD	21.4±3	20.6±3.4	18.3±2.8	20.4±3.6
	Group O	Mean±SD	21.6±3.6	20.3±3.5	19.6±3.1	22±3.6
	Student’s t-test	t	-0.203	0.387	-1.171	-0.705
		P	0.839	0.7	0.253	0.501
Lactate	Group N	Mean±SD	2±1.1	2.3±1.1	2.5±0.9	2.6±0.6
	Group O	Mean±SD	1.7±0.9	2.2±1.1	2.2±0.8	1.9±0.6
	Student’s t-test	t	1.343	0.654	1.028	1.947
		P	0.183	0.515	0.314	0.087
BD	Group N	Mean±SD	-2.6±3	-5±2.1	-6.1±1.2	-6±0.7
	Group O	Mean±SD	-2.6±3.8	-5.2±2.6	-5.8±1.1	-5.5±0.8
	Student’s t-test	t	-0.09	0.294	-0.817	-1.234
		P	0.928	0.769	0.421	0.252

SjvO₂ showed a statistically significant difference at T2 (p < 0.05), with Group O showing higher saturation. A second significant difference was noted in PVO₂ at T4 (p < 0.05), favoring Group O. These isolated findings may indicate transient differences in cerebral oxygen utilization or delivery but were not consistent across other time points.

Lactate levels showed a gradual rise in Group N, indicating increased metabolic demand or reduced clearance, while Group O showed a less consistent pattern. Base deficit decreased in both groups over time, reflecting metabolic recovery. Venous pH remained stable in Group N but showed a slight decline in Group O. SVO₂ increased in both groups, though only T2 showed a significant difference. PVO₂ and PVCO₂ decreased from T1 to T4, but these changes were not significant, aside from the PVO₂ value at T4 (Table 4).

**Table 4:** Intergroup comparison of central venous gas values pH: potential of hydrogen; SvO_2_: mixed venous oxygen saturation; PvO₂: partial pressure of oxygen in mixed venous blood; PvCO_2_: partial pressure of carbon dioxide in mixed venous blood; HCO3: bicarbonate; BD: base deficit; T: time after surgery (hourly); Group N: new blood, received PRBCs stored for less than or equal to 14 days; Group O: old blood, received PRBCs stored for more than 14 days; PRBCs: packed red blood cells

Variables			T1	T2	T3	T4
pH	Group N	Mean±SD	7.34±0.0	7.32±0.0	7.35±0.0	7.33±0.0
	Group O	Mean±SD	7.41±0.1	7.33±0.1	7.31±0.0	7.35±1.8
	Student’s t-test	t	-1.022	-0.315	-0.527	1.003
		P	0.31	0.754	0.602	0.345
SvO_2_	Group N	Mean±SD	79.9±5.4	84.2±2.8	84.1±2.6	85.7±1.1
	Group O	Mean±SD	81.7±5	82.3±4.9	82.4±3.3	82±4.6
	Student’s t-test	t	-1.587	2.176	1.427	1.719
		P	0.117	0.033	0.165	0.124
PvO₂	Group N	Mean±SD	54.1±6.5	51.1±4.3	49.5±5	45.6±5.7
	Group O	Mean±SD	56.1±5	51.7±4.4	51.1±4.1	53.7±1.7
	Student’s t-test	t	-1.533	-0.658	-0.906	-3.336
		P	0.129	0.512	0.373	0.009
PvCO_2_	Group N	Mean±SD	40.8±3.4	40.6±4.8	38.8±4.7	37.5±1.4
	Group O	Mean±SD	41.2±3.6	41.3±3.9	40±4.5	38±3.2
	Student’s t-test	t	-0.467	-0.661	-0.717	-0.319
		P	0.642	0.51	0.48	0.758
HCO3	Group N	Mean±SD	20.7±2.4	20±2.7	18.3±2.8	20.4±3.6
	Group O	Mean±SD	20.5±2.7	19.9±2.8	19.3±2.6	20.7±2.8
	Student’s t-test	t	0.435	0.084	-0.987	-0.156
		P	0.665	0.933	0.333	0.88
Lactate	Group N	Mean±SD	2.1±1.1	2.4±1.2	2.6±0.9	2.6±0.6
	Group O	Mean±SD	1.8±1	2.1±1.1	2.2±0.8	1.9±0.6
	Student’s t-test	t	1.475	1.134	1.19	1.642
		P	0.144	0.26	0.246	0.139
BD	Group N	Mean±SD	-2.7±3.1	-5.1±2.1	-6.2±1.1	-6.2±0.5
	Group O	Mean±SD	-2.9±3.5	-4.9±2.7	-5.7±1.2	-5.4±0.8
	Student’s t-test	t	0.32	-0.42	-1.139	-1.655
		P	0.75	0.675	0.266	0.136

Clinical outcomes

Group N exhibited slightly shorter durations of ICU and hospital stay compared to Group O. However, these differences did not achieve statistical significance (p > 0.05). Functional outcomes, assessed by the GOSE, were also similar between the two groups at discharge, suggesting comparable overall recovery. The decision to transfuse blood has to be made intraoperatively and after enrollment in the study (Table 1).

## Discussion

Craniotomy and excision of intracranial tumors often lead to significant blood loss, which can cause cerebral ischemia and necessitate blood transfusions [1,2]. The transfusion of PRBCs, particularly those stored for prolonged durations, may influence erythrocyte oxygen affinity, deformability, membrane stability, and overall function, factors critical to transfusion efficacy [7-10]. Despite these concerns, there remains no consensus on the optimal cutoff age defining “fresh” versus “old” PRBCs, as the clinical impact of storage lesions remains debated [14]. In our study, we assessed the association between PRBC storage age and cerebral oxygenation alongside neurological outcomes in patients undergoing elective cranial surgery.

Our demographic analysis revealed comparable mean ages between the new blood group (36.75±11.96 years) and the old blood group (40.45±12.25 years), with no statistically significant differences in gender distribution. This aligns with findings from Xiao et al., who reported similar age distributions and male predominance across fresh and old RBC groups in isolated TBI cases [5]. Similarly, Bishnoi et al. and Dhabangi et al. observed no significant demographic differences in pediatric cardiac surgery and severe anemia cases, respectively [14, 17].

Intraoperative parameters such as surgery duration, blood loss, and transfusion volumes of PRBCs, FFP, and platelets were comparable between groups. These findings mirror those by Xiao et al., who reported no differences in pretransfusion hemoglobin levels, RBC transfusion volumes, or additional blood component usage between groups [5]. Likewise, Bishnoi et al. and Dhabangi et al. reported similar transfusion profiles [14,17].

Hemodynamic parameters like HR, SBP, DBP, CVP, body temperature, and SaO2 were largely similar throughout surgery, except for a significantly higher MAP in the old blood group during early intraoperative periods (T1 to T6).

Arterial blood gas analyses, including pH, oxygenation, carbon dioxide levels, HCO3, lactate, and base deficit, showed no significant intergroup differences. These findings corroborate previous studies indicating minimal physiological disturbances attributable to PRBC storage age [17-19]. The concept of the "storage lesion," a spectrum of biochemical, morphological, and rheological changes occurring in stored RBCs, may impair microvascular perfusion due to decreased adenosine triphosphate (ATP), membrane instability, increased potassium, lactate accumulation, and hemolysis. [7-11] Despite these well-documented alterations, our comparative analyses of jugular venous and systemic parameters such as pH, SaO_2_, PaO_2_, PaCO_2_, HCO3, lactate, and base deficit revealed no significant differences except transient changes in PvO_2_ and SvO_2_ at select time points.

Notably, studies by Yamal et al. [15] similarly found no correlation between RBC storage age and cerebral oxygenation parameters or long-term neurological outcomes after TBI. Consistent with these observations, our study showed no significant differences in ICU or hospital length of stay or GOSE scores between groups. These outcomes reflect findings by van de Watering et al. and other investigators who reported no association between RBC storage duration and intensive care or hospital stay length [20-23].

Contrastingly, some trauma studies have suggested increased mortality with transfusion of RBCs stored beyond 14 days, particularly in patients receiving large transfusion volumes [24, 25]. However, our findings align with analyses by Xiao et al. and Ruel-Laliberte et al., which demonstrated no improvement in neurological outcomes with fresher blood in TBI patients [5, 26].

Limitations of the study included a relatively small sample size, and cerebral oximetry monitoring was restricted to intraoperative jugular venous oxygen saturation without supplementary modalities like near-infrared spectroscopy (NIRS). The amount of blood transfused is small (average of two units per patient), and the mean duration of blood storage was not compared.

Future research should include larger, randomized controlled trials encompassing high-risk patients, those requiring greater transfusion volumes, and individuals with significant preoperative comorbidities. Incorporating advanced cerebral oximetry techniques such as NIRS could provide more comprehensive insights into cerebral oxygenation dynamics related to PRBC storage age.

## Conclusions

In this study of adult patients undergoing elective craniotomy, transfusion of RBCs stored for longer durations showed no significant impact on hemodynamic stability, cerebral tissue oxygenation, or neurological outcomes compared to fresher units. Furthermore, no differences were observed in ICU or hospital length of stay between groups. These results suggest that the use of older PRBCs is safe and does not adversely affect perioperative or long-term outcomes in this patient population. Larger randomized trials are warranted to further validate these findings.

## References

[1] Ali Z, Hassan N, Syed S (2014). Blood transfusion practices in neuroanaesthesia. Indian J Anaesth.

[2] McEwen J, Huttunen KH (2009). Transfusion practice in neuroanesthesia. Curr Opin Anaesthesiol.

[3] Bydon M, Abt NB, Macki M, Brem H, Huang J, Bydon A, Tamargo RJ (2014). Preoperative anemia increases postoperative morbidity in elective cranial neurosurgery. Surg Neurol Int.

[4] Mairbäurl H (1994). Red blood cell function in hypoxia at altitude and exercise. Int J Sports Med.

[5] Xiao K, Zhao F, Liu Q (2020). Effect of red blood cell storage duration on outcomes of isolated traumatic brain injury. Med Sci Monit.

[6] Dhar R, Zazulia AR, Videen TO, Zipfel GJ, Derdeyn CP, Diringer MN (2009). Red blood cell transfusion increases cerebral oxygen delivery in anemic patients with subarachnoid hemorrhage. Stroke.

[7] Chin-Yee I, Arya N, d'Almeida MS (1997). The red cell storage lesion and its implication for transfusion. Transfus Sci.

[8] Lelubre C, Vincent JL (2013). Relationship between red cell storage duration and outcomes in adults receiving red cell transfusions: a systematic review. Crit Care.

[9] Hod EA, Spitalnik SL (2011). Harmful effects of transfusion of older stored red blood cells: iron and inflammation. Transfusion.

[10] Hod EA, Brittenham GM, Billote GB (2011). Transfusion of human volunteers with older, stored red blood cells produces extravascular hemolysis and circulating non-transferrin-bound iron. Blood.

[11] Silliman CC, Voelkel NF, Allard JD, Elzi DJ, Tuder RM, Johnson JL, Ambruso DR (1998). Plasma and lipids from stored packed red blood cells cause acute lung injury in an animal model. J Clin Invest.

[12] Lelubre C, Vincent JL (2011). Red blood cell transfusion in the critically ill patient. Ann Intensive Care.

[13] Hess JR (2006). An update on solutions for red cell storage. Vox Sang.

[14] Bishnoi AK, Garg P, Patel K (2019). Effect of red blood cell storage duration on outcome after paediatric cardiac surgery: a prospective observational study. Heart Lung Circ.

[15] Yamal JM, Benoit JS, Doshi P, Rubin ML, Tilley BC, Hannay HJ, Robertson CS (2015). Association of transfusion red blood cell storage age and blood oxygenation, long-term neurologic outcome, and mortality in traumatic brain injury. J Trauma Acute Care Surg.

[16] Aubron C, Bailey M, McQuilten Z (2014). Duration of red blood cells storage and outcome in critically ill patients. J Crit Care.

[17] Dhabangi A, Ainomugisha B, Cserti-Gazdewich C (2015). Effect of transfusion of red blood cells with longer vs shorter storage duration on elevated blood lactate levels in children with severe anemia: the TOTAL randomized clinical trial. JAMA.

[18] Keidan I, Amir G, Mandel M, Mishali D (2004). The metabolic effects of fresh versus old stored blood in the priming of cardiopulmonary bypass solution for pediatric patients. J Thorac Cardiovasc Surg.

[19] Kopterides P, Theodorakopoulou M, Nikitas N (2012). Red blood cell transfusion affects microdialysis-assessed interstitial lactate/pyruvate ratio in critically ill patients with late sepsis. Intensive Care Med.

[20] van de Watering L, Lorinser J, Versteegh M, Westendord R, Brand A (2006). Effects of storage time of red blood cell transfusions on the prognosis of coronary artery bypass graft patients. Transfusion.

[21] Yap CH, Lau L, Krishnaswamy M, Gaskell M, Yii M (2008). Age of transfused red cells and early outcomes after cardiac surgery. Ann Thorac Surg.

[22] McKenny M, Ryan T, Tate H, Graham B, Young VK, Dowd N (2011). Age of transfused blood is not associated with increased postoperative adverse outcome after cardiac surgery. Br J Anaesth.

[23] Sanders J, Patel S, Cooper J, Berryman J, Farrar D, Mythen M, Montgomery HE (2011). Red blood cell storage is associated with length of stay and renal complications after cardiac surgery. Transfusion.

[24] Weinberg JA, McGwin G Jr, Griffin RL (2008). Age of transfused blood: an independent predictor of mortality despite universal leukoreduction. J Trauma.

[25] Spinella PC, Carroll CL, Staff I (2009). Duration of red blood cell storage is associated with increased incidence of deep vein thrombosis and in hospital mortality in patients with traumatic injuries. Crit Care.

[26] Ruel-Laliberté J, Lessard Bonaventure P, Fergusson D (2019). Effect of age of transfused red blood cells on neurologic outcome following traumatic brain injury (ABLE-tbi Study): a nested study of the Age of Blood Evaluation (ABLE) trial. Can J Anaesth.

